# Diaqua­bis[5-(2-pyridylmeth­yl)tetra­zolato-κ^2^ *N* ^1^,*N* ^5^]manganese(II)

**DOI:** 10.1107/S1600536808019272

**Published:** 2008-07-05

**Authors:** Wei Wang

**Affiliations:** a Ordered Matter Science Research Center, Southeast University, Nanjing 210096, People’s Republic of China

## Abstract

The title complex, [Mn(C_7_H_6_N_5_)_2_(H_2_O)_2_], was obtained by the *in situ* hydro­thermal reaction of MnCl_2_ with 2-(2-pyrid­yl)acetonitrile in the presence of NaN_3_. The Mn^II^ atom, which is located on an inversion centre, has a distorted octa­hedral coordination geometry formed by two water mol­ecules and two chelating ligands. Inter­molecular hydrogen bonds and π–π inter­actions (3.452 Å) stabilize the crystal structure and lead to the formation of a three-dimensional network.

## Related literature

For related literature, see: Demko & Sharpless (2001 ▶); Zhao *et al.* (2008 ▶). For the synthesis of similar complexes, see: Hu *et al.* (2007 ▶); Liu & Fan (2007 ▶).
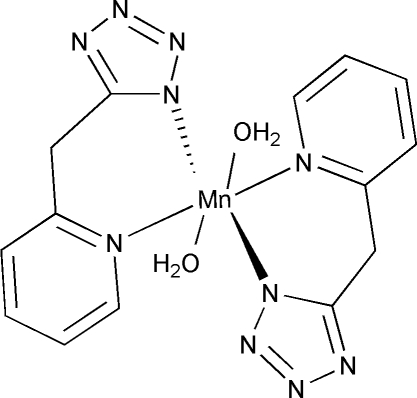


## Experimental

### 

#### Crystal data

[Mn(C_7_H_6_N_5_)_2_(H_2_O)_2_]
*M* *_r_* = 411.31
Monoclinic, 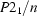

*a* = 6.638 (2) Å
*b* = 13.788 (5) Å
*c* = 8.771 (3) Å
β = 90.01 (5)°
*V* = 802.9 (4) Å^3^
*Z* = 2
Mo *K*α radiation
μ = 0.86 mm^−1^
*T* = 293 (2) K
0.20 × 0.12 × 0.12 mm

#### Data collection

Rigaku Mercury2 diffractometer
Absorption correction: multi-scan (*CrystalClear*; Rigaku, 2005 ▶) *T*_min_ = 0.802, *T*_max_ = 1.000 (expected range = 0.723–0.902)
8070 measured reflections
1836 independent reflections
1550 reflections with *I* > 2σ(*I*)
*R*_int_ = 0.057

#### Refinement

*R*[*F* ^2^ > 2σ(*F* ^2^)] = 0.058
*wR*(*F* ^2^) = 0.172
*S* = 1.13
1836 reflections
124 parameters
H-atom parameters constrained
Δρ_max_ = 0.39 e Å^−3^
Δρ_min_ = −0.73 e Å^−3^

### 

Data collection: *CrystalClear* (Rigaku, 2005 ▶); cell refinement: *CrystalClear*; data reduction: *CrystalClear*; program(s) used to solve structure: *SHELXS97* (Sheldrick, 2008 ▶); program(s) used to refine structure: *SHELXL97* (Sheldrick, 2008 ▶); molecular graphics: *SHELXTL* (Sheldrick, 2008 ▶); software used to prepare material for publication: *SHELXTL*.

## Supplementary Material

Crystal structure: contains datablocks I, global. DOI: 10.1107/S1600536808019272/hg2418sup1.cif

Structure factors: contains datablocks I. DOI: 10.1107/S1600536808019272/hg2418Isup2.hkl

Additional supplementary materials:  crystallographic information; 3D view; checkCIF report

## Figures and Tables

**Table 1:** Hydrogen-bond geometry (Å, °)

*D*—H⋯*A*	*D*—H	H⋯*A*	*D*⋯*A*	*D*—H⋯*A*
O1—H1*B*⋯N2^i^	0.96	2.04	2.889 (8)	146
O1—H1*B*⋯N5^i^	0.96	2.45	3.371 (8)	162
O1—H1*C*⋯N4^ii^	0.96	1.96	2.786 (8)	142
C6—H6*A*⋯N5^iii^	0.97	2.60	3.343 (5)	133

Symmetry codes: (i) 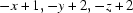; (ii) 
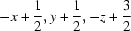
; (iii) 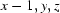.

**Fig. 1.**
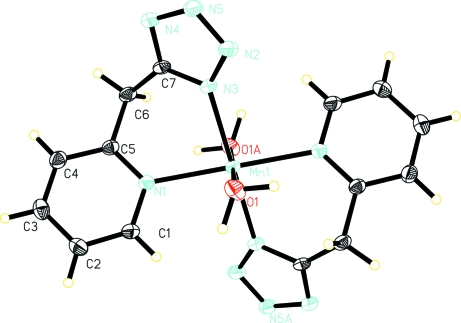
The molecular structure of the compound with the atomic numbering scheme. Displacement ellipsoids were drawn at the 30% probability level. [symmetry code: -x, -y+2, -z+2]

**Fig. 2.**
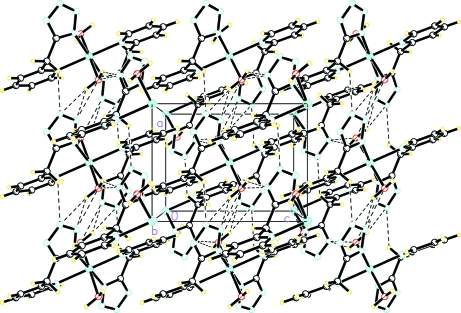
The packing view of title compound with π···π stacking along the b axis.

## supplementary crystallographic information

### Comment

Since Sharpless *et al.* reported the environmentally friendly process for the preparation of tetrazole (Demko & Sharpless, 2001), many novel tetrazole compounds have been reported through 2 + 3 cycloaddition reactions. Work in our group have found that single crystals of coordination polymers can often be generated under hydrothermal conditions through *in situ* synthesis. (Zhao *et al.*, 2008) The title complex was obtained by the *in situ* hydrothermal reaction of MnCl_2_ with pyridin-2-yl-acetonitrile in the presence of NaN_3_.

In the title compound, the central Mn(II) ion is located on an inversion center and coordinated by two water molecules and two 5-(pyridin-2-ylmethyl)tetrazolate ligands through the pyridine N and tetrazole N atoms with a distorted octahedral geometry (Fig. 1). Extensive intermolecular O—H···N and C—H···N hydrogen bonds and π-π interactions stabilize the crystal structure which leads to the formation of a three-dimensional network.

### Experimental

A mixture of pyridin-2-yl-acetonitrile (26 mg, 0.2 mmol), NaN_3_ (26 mg, 0.4 mmol), MnCl_2_.4H_2_O(59.3 mg, 0.3 mmol), ethanol (1 ml) and a few drops of water sealed in a glass tube was maintained at 105°C. Colorless crystals suitable for X-ray analysis were obtained after a week.

### Refinement

The C-bound H atoms were placed in calculated positions (C—H 0.93 Å) and treated in the subsequent refinement as riding atoms, with *U*_iso_(H) = 1.2*U*_eq_(C) while the water H atoms were located in Fourier difference map and refined with *U*_iso_(H) = 1.5*U*_eq_(O).

### Crystal data

**Table:**

[Mn(C_7_H_6_N_5_)_2_(H_2_O)_2_]	*F*_000_ = 422
*M**_r_* = 411.31	*D*_x_ = 1.701 Mg m^−^^3^
Monoclinic, *P*2_1_/*n*	Mo *K*α radiation λ = 0.71073 Å
Hall symbol: -P 2yn	Cell parameters from 2050 reflections
*a* = 6.639 (2) Å	θ = 2.8–27.5º
*b* = 13.788 (5) Å	µ = 0.86 mm^−^^1^
*c* = 8.771 (3) Å	*T* = 293 (2) K
β = 90.01 (5)º	Prism, colorless
*V* = 802.9 (4) Å^3^	0.20 × 0.12 × 0.12 mm
*Z* = 2	

### Data collection

**Table:**

Rigaku Mercury2) diffractometer	1836 independent reflections
Radiation source: fine-focus sealed tube	1550 reflections with *I* > 2σ(*I*)
Monochromator: graphite	*R*_int_ = 0.057
Detector resolution: 13.6612 pixels mm^-1^	θ_max_ = 27.5º
*T* = 293(2) K	θ_min_ = 3.0º
CCD_Profile_fitting scans	*h* = −8→8
Absorption correction: multi-scan(CrystalClear; Rigaku, 2005)	*k* = −17→17
*T*_min_ = 0.802, *T*_max_ = 1.000	*l* = −11→11
8070 measured reflections	

### Refinement

**Table:**

Refinement on *F*^2^	Secondary atom site location: difference Fourier map
Least-squares matrix: full	Hydrogen site location: inferred from neighbouring sites
*R*[*F*^2^ > 2σ(*F*^2^)] = 0.058	H-atom parameters constrained
*wR*(*F*^2^) = 0.173	*w* = 1/[σ^2^(*F*_o_^2^) + (0.0834*P*)^2^ + 0.8368*P*] where *P* = (*F*_o_^2^ + 2*F*_c_^2^)/3
*S* = 1.13	(Δ/σ)_max_ < 0.001
1836 reflections	Δρ_max_ = 0.39 e Å^−^^3^
124 parameters	Δρ_min_ = −0.73 e Å^−^^3^
Primary atom site location: structure-invariant direct methods	Extinction correction: none

### Special details

**Table:**

Geometry. All e.s.d.'s (except the e.s.d. in the dihedral angle between two l.s. planes) are estimated using the full covariance matrix. The cell e.s.d.'s are taken into account individually in the estimation of e.s.d.'s in distances, angles and torsion angles; correlations between e.s.d.'s in cell parameters are only used when they are defined by crystal symmetry. An approximate (isotropic) treatment of cell e.s.d.'s is used for estimating e.s.d.'s involving l.s. planes.
Refinement. Refinement of *F*^2^ against ALL reflections. The weighted *R*-factor *wR* and goodness of fit *S* are based on *F*^2^, conventional *R*-factors *R* are based on *F*, with *F* set to zero for negative *F*^2^. The threshold expression of *F*^2^ > σ(*F*^2^) is used only for calculating *R*-factors(gt) *etc*. and is not relevant to the choice of reflections for refinement. *R*-factors based on *F*^2^ are statistically about twice as large as those based on *F*, and *R*- factors based on ALL data will be even larger.

### Fractional atomic coordinates and isotropic or equivalent isotropic displacement parameters (Å^2^)

**Table:**

	*x*	*y*	*z*	*U*_iso_*/*U*_eq_	
Mn1	0.0000	1.0000	1.0000	0.0261 (3)	
N5	0.4367 (4)	0.7962 (2)	0.8387 (3)	0.0354 (7)	
O1	0.2353 (4)	1.10029 (18)	0.9172 (3)	0.0382 (6)	
H1B	0.3241	1.1164	0.9997	0.057*	
H1C	0.1730	1.1583	0.8792	0.057*	
N3	0.1879 (4)	0.88112 (19)	0.9162 (3)	0.0299 (6)	
C7	0.1284 (5)	0.8238 (2)	0.8048 (4)	0.0275 (7)	
N2	0.3843 (4)	0.8625 (2)	0.9339 (3)	0.0346 (7)	
N4	0.2778 (4)	0.7701 (2)	0.7545 (3)	0.0332 (6)	
C6	−0.0793 (5)	0.8223 (2)	0.7415 (4)	0.0317 (7)	
H6A	−0.1720	0.8028	0.8214	0.038*	
H6B	−0.0860	0.7738	0.6614	0.038*	
C5	−0.1465 (5)	0.9175 (2)	0.6782 (4)	0.0289 (7)	
C4	−0.2173 (5)	0.9233 (3)	0.5316 (4)	0.0359 (8)	
H4A	−0.2262	0.8677	0.4720	0.043*	
C3	−0.2743 (6)	1.0105 (3)	0.4737 (4)	0.0371 (8)	
H3A	−0.3202	1.0155	0.3738	0.045*	
C2	−0.2628 (6)	1.0907 (3)	0.5649 (4)	0.0377 (8)	
H2A	−0.3022	1.1513	0.5290	0.045*	
C1	−0.1925 (5)	1.0797 (2)	0.7089 (4)	0.0340 (8)	
H1A	−0.1832	1.1345	0.7704	0.041*	
N1	−0.1363 (4)	0.99533 (17)	0.7671 (3)	0.0273 (6)	

### Atomic displacement parameters (Å^2^)

**Table:**

	*U*^11^	*U*^22^	*U*^33^	*U*^12^	*U*^13^	*U*^23^
Mn1	0.0329 (4)	0.0185 (4)	0.0270 (4)	0.0025 (2)	−0.0001 (3)	−0.0016 (2)
N5	0.0353 (15)	0.0297 (15)	0.0413 (16)	0.0058 (12)	−0.0018 (12)	−0.0047 (12)
O1	0.0381 (13)	0.0326 (13)	0.0439 (14)	−0.0103 (10)	−0.0061 (11)	0.0116 (11)
N3	0.0341 (15)	0.0230 (13)	0.0326 (14)	0.0049 (11)	−0.0004 (11)	−0.0030 (11)
C7	0.0324 (16)	0.0155 (13)	0.0345 (16)	0.0003 (11)	0.0025 (13)	−0.0007 (12)
N2	0.0328 (15)	0.0265 (14)	0.0446 (16)	0.0041 (11)	−0.0027 (12)	−0.0019 (12)
N4	0.0371 (15)	0.0253 (13)	0.0371 (16)	0.0054 (11)	−0.0003 (12)	−0.0037 (12)
C6	0.0339 (17)	0.0217 (15)	0.0395 (17)	−0.0018 (12)	−0.0022 (14)	−0.0058 (13)
C5	0.0271 (15)	0.0245 (15)	0.0349 (17)	−0.0007 (12)	−0.0006 (13)	−0.0032 (13)
C4	0.0334 (17)	0.0372 (19)	0.0373 (18)	0.0010 (14)	−0.0045 (14)	−0.0081 (15)
C3	0.0299 (18)	0.051 (2)	0.0303 (17)	−0.0013 (14)	−0.0013 (14)	0.0029 (15)
C2	0.0399 (19)	0.0357 (18)	0.0376 (18)	0.0039 (15)	−0.0015 (15)	0.0079 (15)
C1	0.0417 (19)	0.0246 (16)	0.0357 (17)	0.0038 (13)	0.0001 (14)	0.0003 (13)
N1	0.0289 (14)	0.0239 (14)	0.0291 (14)	0.0009 (9)	0.0012 (11)	0.0003 (10)

### Geometric parameters (Å, °)

**Table:**

Mn1—N3	2.187 (5)	C6—H6A	0.9700
Mn1—O1	2.209 (5)	C6—H6B	0.9700
Mn1—N1	2.235 (3)	C5—N1	1.328 (5)
N5—N2	1.286 (5)	C5—C4	1.371 (6)
N5—N4	1.337 (5)	C4—C3	1.359 (6)
O1—H1B	0.9600	C4—H4A	0.9300
O1—H1C	0.9600	C3—C2	1.367 (6)
N3—C7	1.317 (5)	C3—H3A	0.9300
N3—N2	1.338 (6)	C2—C1	1.355 (6)
C7—N4	1.314 (5)	C2—H2A	0.9300
C7—C6	1.487 (6)	C1—N1	1.324 (5)
C6—C5	1.494 (6)	C1—H1A	0.9300
			
N3—Mn1—O1	87.43 (11)	C7—C6—H6B	108.8
N3^i^—Mn1—O1	92.57 (5)	C5—C6—H6B	108.8
N3—Mn1—N1	84.39 (17)	H6A—C6—H6B	107.7
N3^i^—Mn1—N1	95.61 (17)	N1—C5—C4	121.4 (3)
O1^i^—Mn1—N1	89.79 (18)	N1—C5—C6	118.5 (4)
O1—Mn1—N1	90.21 (18)	C4—C5—C6	120.1 (3)
N2—N5—N4	109.6 (3)	C3—C4—C5	119.9 (3)
Mn1—O1—H1B	109.3	C3—C4—H4A	120.1
Mn1—O1—H1C	109.3	C5—C4—H4A	120.1
H1B—O1—H1C	109.5	C4—C3—C2	118.8 (4)
C7—N3—N2	105.3 (3)	C4—C3—H3A	120.6
C7—N3—Mn1	121.9 (3)	C2—C3—H3A	120.6
N2—N3—Mn1	131.4 (2)	C1—C2—C3	118.3 (4)
N3—C7—N4	111.1 (3)	C1—C2—H2A	120.9
N3—C7—C6	124.3 (3)	C3—C2—H2A	120.9
N4—C7—C6	124.5 (3)	N1—C1—C2	123.7 (3)
N5—N2—N3	109.0 (3)	N1—C1—H1A	118.2
C7—N4—N5	105.0 (3)	C2—C1—H1A	118.2
C7—C6—C5	113.8 (3)	C1—N1—C5	118.0 (4)
C7—C6—H6A	108.8	C1—N1—Mn1	116.2 (2)
C5—C6—H6A	108.8	C5—N1—Mn1	125.5 (2)
			
O1^i^—Mn1—N3—C7	64.4 (3)	C7—C6—C5—C4	125.9 (3)
O1—Mn1—N3—C7	−115.6 (3)	N1—C5—C4—C3	1.5 (5)
N1—Mn1—N3—C7	−25.2 (3)	C6—C5—C4—C3	−178.6 (3)
O1^i^—Mn1—N3—N2	−131.9 (3)	C5—C4—C3—C2	−1.1 (6)
O1—Mn1—N3—N2	48.1 (3)	C4—C3—C2—C1	0.8 (6)
N1—Mn1—N3—N2	138.5 (3)	C3—C2—C1—N1	−0.8 (6)
N1^i^—Mn1—N3—N2	−41.5 (3)	C2—C1—N1—C5	1.1 (5)
N2—N3—C7—N4	0.8 (4)	C2—C1—N1—Mn1	174.6 (3)
Mn1—N3—C7—N4	168.2 (2)	C4—C5—N1—C1	−1.5 (5)
N2—N3—C7—C6	−177.6 (3)	C6—C5—N1—C1	178.7 (3)
Mn1—N3—C7—C6	−10.2 (4)	C4—C5—N1—Mn1	−174.3 (2)
N4—N5—N2—N3	0.7 (4)	C6—C5—N1—Mn1	5.9 (4)
C7—N3—N2—N5	−0.9 (4)	N3—Mn1—N1—C1	−145.1 (3)
Mn1—N3—N2—N5	−166.6 (2)	N3^i^—Mn1—N1—C1	34.9 (3)
N3—C7—N4—N5	−0.3 (4)	O1^i^—Mn1—N1—C1	122.3 (3)
C6—C7—N4—N5	178.0 (3)	O1—Mn1—N1—C1	−57.7 (3)
N2—N5—N4—C7	−0.3 (4)	N3—Mn1—N1—C5	27.8 (3)
N3—C7—C6—C5	59.0 (5)	N3^i^—Mn1—N1—C5	−152.2 (3)
N4—C7—C6—C5	−119.2 (4)	O1^i^—Mn1—N1—C5	−64.8 (3)
C7—C6—C5—N1	−54.2 (4)	O1—Mn1—N1—C5	115.2 (3)

Symmetry codes: (i) −*x*, −*y*+2, −*z*+2.

### Hydrogen-bond geometry (Å, °)

**Table:**

*D*—H···*A*	*D*—H	H···*A*	*D*···*A*	*D*—H···*A*
O1—H1B···N2^ii^	0.96	2.04	2.889 (8)	146
O1—H1B···N5^ii^	0.96	2.45	3.371 (8)	162
O1—H1C···N4^iii^	0.96	1.96	2.786 (8)	142
C6—H6A···N5^iv^	0.97	2.60	3.343 (5)	133

Symmetry codes: (ii) −*x*+1, −*y*+2, −*z*+2; (iii) −*x*+1/2, *y*+1/2, −*z*+3/2; (iv) *x*−1, *y*, *z*.
